# Professionals’ perspectives on existing practice and conditions for nurse-led gout care based on treatment recommendations: a qualitative study in primary healthcare

**DOI:** 10.1186/s12875-022-01677-z

**Published:** 2022-04-07

**Authors:** Helene Sedelius, Malin Tistad, Ulrika Bergsten, Mats Dehlin, David Iggman, Lars Wallin, Anna Svärd

**Affiliations:** 1 School of Health and Welfare, Dalarna University, Falun, Sweden; 2 Centre for Clinical Research Dalarna-Uppsala University, Falun, Sweden; 3 Department of Neurobiology, Care Sciences and Society, Karolinska Institute, Stockholm, Sweden; 4 R&D Department at Region Halland, Halmstad, Sweden; 5 Department of Rheumatology and Inflammation Research, Institution of Medicine, Sahlgrenska Academy, University of Gothenburg SE, Gothenburg, Sweden

**Keywords:** Gout, Primary healthcare, Personnel experiences, Experiences, Implementation, I-PARIHS, Nurse-led care

## Abstract

**Background:**

Gout affects nearly 2 % of the population and is associated with repeated painful flares of arthritis. Preventive urate-lowering therapy is widely available, but only one third of patients receive adequate treatment. Lack of knowledge among healthcare professionals and patients within primary healthcare are implicated as partial explanations for this undertreatment. Nurse-led care has proved to be an effective model when treating patients with gout, but there is a need for more knowledge about factors that can be expected to influence the future implementation of such care. The aim of this study was to describe factors influencing existing gout care in primary healthcare and the conditions for a future implementation of nurse-led gout care based on national treatment recommendations.

**Methods:**

In this qualitative study, focus group discussions with 56 nurses and physicians and individual interviews with eight managers were conducted at nine primary healthcare units in central Sweden. A deductive qualitative content analysis based on the main constructs of the framework Integrated Promoting Action on Research Implementation in Health Services was followed by an inductive analysis within the frames of the main constructs: innovation, recipients and context.

**Results:**

Gout-related contacts with primary healthcare was described as being patient initiated, diagnostics was in some respects complex and nurse-led care was experienced as a favourable primary healthcare model in general (innovation). Gout was seen as a low-priority condition with acute flares and there was inadequate knowledge of gout, including preventive treatment (recipients). Primary healthcare was perceived as having a holistic but fragmented responsibility for gout care, recommendations against keeping waiting lists complicated follow-up appointments and a need for motivation and support when introducing new practices was emphasised (context).

**Conclusion:**

In this study, investigating the perspective of professionals, several factors were found to influence existing gout care. It will be crucial to target these factors in the development of a future implementation strategy.

**Supplementary Information:**

The online version contains supplementary material available at 10.1186/s12875-022-01677-z.

## Background

Gout is becoming more common, partly due to increasing age and unhealthy living habits in the population [1]. It affects 1.8% of the adult population of Sweden, with the majority being men [2]. The disease is caused by high levels of urate in the blood, which can lead to formation of monosodium urate crystals in the joints, commonly a peripheral joint in the foot, giving rise to a typical gout flare [3]. It is characterised by severe inflammation and pain caused by an immunological reaction to the urate crystals. At the onset of disease, the gout flares are recurrent but self-limiting. Without urate lowering treatment (ULT) to dissolve the urate crystals, flares tend to come more frequent and the disease may develop into a chronic state of inflammation and pain, chronic gouty arthritis. In relation to this, destruction of affected joints and tophi, deposits of urate crystals usually in or around the joints, are often seen [4]. Premature mortality is increased for patients with gout and to a large degree this can be explained by comorbidities such as hypertension, hyperlipidaemia, chronic kidney disease, obesity, diabetes [5] and cardiovascular disease in particular [6]. In addition, gout adversely affects health-related quality of life [7]. Even though preventive drug treatments are available, inexpensive and effective, only a third of individuals with gout receive adequate pharmacological treatment in Sweden and the UK [8, 9]. There are indications that inadequate knowledge among physicians and nurses [10], general lack of primary healthcare (PHC) resources and insufficient knowledge and motivation among patients [11] are key factors contributing to the low frequency of treatment.

To improve the management of gout, the first Swedish treatment recommendations were published in 2016 [12]. They consist of both pharmacological and non-pharmacological treatment, as described in Table 1. According to these recommendations, the dose of ULT should be increased stepwise until the target levels of urate is reached, a so-called “treat-to-target titration”. The expression “gout care” will henceforth be used for the combination of pharmacological and non-pharmacological care and treatment. Most patients with gout are treated in PHC, which is also the first point of contact for patients in the Swedish healthcare system. All private and public PHC units in Sweden are publicly funded and are expected to provide the same services to their enrolled patients [13].

**Table 1:** Swedish treatment recommendations [12]

**Pharmacological treatment** One gout flare in combination with at least one of the following risk factors for recurrent disease requires long-term pharmacological urate lowering treatment (ULT), sometimes life-long:	
• age below 40 years	
• urate level > 480 μmol/L	
• more than one flare	
• multiple joint engagement	
• skeletal effects	
• comorbidities	
• tophi or urate stone	
ULT should be increased stepwise in dose to achieve target levels of urate; 360 μmol/L in uncomplicated gout and < 300 μmol/L if tophi are present (normal urate levels without gout are in the range 155–480 μmol/L).	
**Non-pharmacological interventions**	
• lifestyle changes, including reduced alcohol consumption	
• appropriate diet	
• weight loss	
• physical activity	
• individualised patient education	

Despite reports on the low adherence to pharmacological gout treatment recommendations [2, 9], only a few randomised controlled trials evaluating organisational models for providing such treatment have been reported [14–16]. In one efficacy study, nurse-led care with a person-centred approach was proven to be beneficial in terms of patients’ adherence to ULT, quality of life and cost effectiveness [16], and with potential to also be favourable when integrated into routine care. However, this model was designed to fit the PHC context in the UK, and adaptations of interventions are often needed when transferred to a different context [17].

Implementation science has identified a number of components that are essential to address in order to accomplish successful implementation of new practices. These components encompass the context where the implementation will be performed, including national and local policies. Knowledge and understanding of the characteristics of the context, the new method, healthcare professionals and patients that might act as barriers or facilitators in a change process are important when strategies for implementation support are being planned and executed [17, 18].

Care according to guidelines, for patients with gout, is provided suboptimally. To reach a deeper understanding of the reasons behind this situation, experiences from a healthcare professional perspective must be examined as well as conditions for the future implementation of treatment recommendations on gout. Consequently, we wanted to increase knowledge about the present situation from the perspective of how professionals view nurse-led gout care as a potential organisational model. This study is the first in a project with the overall goal of improving care and treatment for patients with gout.

The aim of the present study was to describe factors that influence existing gout care in primary healthcare and conditions for the future implementation of nurse-led gout care based on national treatment recommendations.

## Methods

### Design

This study has an explorative descriptive qualitative design [19], applying a content analysis, inspired by Elo and Kyngas [20], on data from focus groups and individual interviews.

### Setting

The healthcare in Sweden is organised in 21 self-governing geographical regions with hospitals and PHC units responsible for healthcare. Physicians and nurses staff PHC units, among other healthcare professionals, and nurse-led care is a well-established way of organising care for chronic diseases such as diabetes and heart failure. The study was conducted at nine PHC units in three healthcare regions in central Sweden, with approximately one million inhabitants altogether. A preliminary number of participating PHC units was set in the beginning. A purposeful selection of units was made out of all units available in order to achieve a broad representation, to obtain as comprehensive information as possible from the interviews. The units were chosen to represent all three included regions, and within the regions different municipalities. Contact details were obtained after the selection process. Initially, five units in one of the regions were recruited. In order to enable more variation in the data, we decided to recruit units in two additional health care regions. In each of these additional regions, two primary care units were included. As no substantial new information emerged in those interviews, we judged that sufficient variation had been achieved and ended recruitment. Both rural (*n* = 5) and non-rural (*n* = 4) areas were included in all regions. One unit was a private corporation, the others were public institutions, but all were publicly funded. The size of the units ranged from 5000 to 21,000 enrolled patients and the number of employees was between 20 and 80.

### Participants

The sample consisted of 56 physicians and nurses and eight managers. A first contact with each PHC unit was made via email and thereafter by phone with the manager, who took a decision whether the PHC unit could participate in the study, everyone asked agreed. The nurses and physicians were recruited in consultation with the managers using a purposeful selection regarding gender and gaining an equal distribution between nurses and physicians. The managers sent an e-mail with the names of the selected participants to the researcher and they then received an invitation to the focus group session by mail, no one declined to participate. All senior managers from the nine units were invited to participate, eight chose to do so. The one who did not had only recently taken up her post and did not have any prior experience of managing a PHC unit, see Table 2 for information about participants per PHC unit. The majority of participants were women, aged between 27 and 66 years, and the mean time working in PHC was 11 years, see Table 3 for detailed information.

**Table 2:** Participants per PHC unit

PHC unit /focus group^**a**^(region)	Nurses (n)	Physicians (n)	Managers (n)
**1 (1)**	5	1	1
**2 (1)**	3	3	1
**3 (1)**	4	4	1
**4 (1)**	2	3	1
**5 (1)**	3	3	1
**6 (2)**	3	3	1
**7 (2)**	2	4	1
**8 (3)**	2	4	0
**9 (3)**	3	4	1

^a^One focus group from each PHC unit

**Table 3:** Information about participants

	Nurses *n* = 27	Physicians *n* = 29	Managers *n* = 8	Total *n* = 64
Age (years) *Mean (range)*	49 (27–65)	47 (30–66)	54 (39–64)	49 (27–66)
Females *n (%)*	24 (89)	11 (38)	8 (100)	43 (67)
Work experience^a^ (years) *Mean (range)*	8 (1–39)	12 (1–34)	17 (5–29)	11 (1–39)

^a^In primary healthcare

### Data collection

Data collection took place at each PHC unit, apart from one interview with a manager conducted over the phone, between February and December 2019. All the nine focus groups were composed of five to eight members, mixed with nurses and physicians and the discussions lasted between 59 and 67 min. The use of focus group interviewing aimed to stimulate and capture a discussion between colleagues and professions [21]. The first author acted as moderator during the focus group discussions. Another member of the research group assisted and took field notes. To our knowledge, the participants did not know any of the researchers. Four main topics served as a guide for the conversation in focus groups: “experiences of gout and patients with gout”, “the care and treatment of patients with gout” , “contextual conditions for, and experiences of, implementation of new practices in general” and “views on and potential of nurse-led care in general (see Additional file 1). These topics were identified as relevant by the research team as they were expected to generate data to achieve the purpose of the study. The moderator prompted participants to explain, freely associate and elaborate on their answers throughout the discussion. A pilot focus group discussion was performed. This did not result in any changes to the four main topics discussed in the main study and was thus included in the analysis. The first author interviewed the eight managers separately due to the assumption that the presence of a manager in a focus group with the employees could influence the discussion. Each interview had a duration of between 25 and 55 min. The questions put to the managers were the same as for the nurses and physicians in the focus group discussions (see Additional file 1). The data collection continued until the information gathered became repetitive and not much new information was being provided by the participants [20], this happened within the preliminary number of interviews set from the beginning. All 17 interviews were held in Swedish thereafter recorded and transcribed verbatim, transcripts were not returned to participants for comments. Information about participants’ age, sex, profession and work experiences was collected using a printed form.

### Data analysis

A content analysis with a deductive approach using an unconstrained matrix, according to Elo and Kyngas [20] was performed. The analysis was based on the main constructs (innovation, recipients and context) of the framework Integrated Promoting Action on Research Implementation in Health Services, i-PARIHS. This framework was considered suitable because it assists in identifying the determinants of the current practice [22], thus enhancing the relevance of the analysis. The i-PARIHS framework was developed based on theory, empirical studies and experiences from quality improvement projects. It provides guidance on what characteristics to consider when the current practice is being explored and when strategies for implementation are being developed. Innovation refers to what is to be implemented, a broad combination of research evidence, knowledge and practice. The recipients capture the perspectives and characteristics of the individuals and teams affected by the innovation (in this study nurses, physicians and managers). The patients’ perspectives, in this analysis, were reflected through all the participants of this study. The construct context in our analysis includes both the PHC units’ inner context, at a local and organisational level, and outer context, representing the wider healthcare system. The construct context in our analysis includes both the PHC units’ inner context, the setting at a local and organisational level, and outer context, representing the wider healthcare, affecting the setting [18] (see Table 4 and additional file 2). Our study examines the present practice and the prerequisites for implementation of nurse-led gout care as a potential organisational model.

**Table 4:** The i-PARIHS’ main constructs, definitions [18]

Innovation	Recipients	Context (and)
What is to be implemented	Perspectives and characteristics of the individuals and teams to be affected by the innovation	Setting for the implementation **-inner** Local and organisational **-outer** **wider healthcare**

The analysis started with listening to the interviews, and the transcripts were thereafter read several times, enabling “units of analysis” to be identified. The identified units were then inductively coded by content and the codes were deductively sorted into the framework i-PARIHS main constructs. The analysis proceeded with analysis of the data under each construct according to the principles of an inductive content analysis, codes were grouped creating sub-categories and from them abstraction to categories as described by Elo and Kyngas [21]. Memos and ideas were noted during the process of analysis. This process implied going back and forth comparing data in relation to the subcategories and categories, as well as the main categories, in between and side by side [21]. The software NVivo 12 Plus was used to handle the data throughout the process.

To achieve credibility, the first author conducted the first readings together with the last and second authors. They identified units of analysis separately, then we compared the identified units and reached consensus on how to select suitable units. There were no major differences between the researchers in the initial coding. Moreover, throughout the process of analysis, continuous discussions were held between the first, second, third and fourth authors to achieve the most valid interpretation of the data. The quotes in the manuscript were at first translated by the authors, the professional translator also received the original quotes in Swedish when doing the language review. Participants did not get opportunities to provide feedback on the findings.

### Ethical considerations

The study was approved by the Swedish Ethical Review Authority: reg. nr. 2019–00077 and 2019–04888. All methods were performed in accordance with the relevant guidelines and regulations. Information about the study’s aim, confidentiality and the participants’ rights was given orally and in writing. All participants signed a consent form, describing the voluntary nature of their participation and their freedom to withdraw at any time and confirmed that confidentiality was guaranteed.

## Results

An overview of the constructs of i-PARIHS and the eight categories identified is outlined in Table 5. An example of the sub-categories of one of the categories is presented in Table 6. An overview of all sub-categories is available in Additional file 3. The results are presented in detail below, according to the analyses of the three constructs and their categories. When no specific group of participants is mentioned, the analysis is valid for all participants.

**Table 5:** An overview of constructs and categories

Constructs	Categories
**Innovation**	Primarily patient-initiated contacts
	Complexity of diagnostics and preventive treatment
	Nurse-led care; a favourable organisational model when time allows
**Recipients**	A low-priority condition with acute flares
	Variations in knowledge about gout and belief in preventive treatment
**Context**, inner and outer	A holistic but fragmented responsibility with limited resources
	Adopting new evidence requires supportive strategies and motivation
	Aggravating circumstances related to systems and recommendations

**Table 6:** An example of a category and its sub-categories

Category	Sub-categories
Complexity of diagnostics and treatment	Clinical practice dependent on physician involved
	A diagnostic dilemma
	Prevention too time-consuming
	Self-care common^a^
	Emphasising the importance of treatment depending on patient preferences^b^

^a^Only expressed by nurses and physicians

^b^Only expressed by managers

### Innovation

#### Primarily patient-initiated contacts

Gout patients were described as primarily initiating the contact themselves and being treated for pain in the acute situation.

All contacts were initiated by the patient and had an acute character due to pain from a gout flare or with another diagnosis in focus. For instance, when patients came for an annual follow-up, due to issues such as heart failure or diabetes, gout and its treatment were discussed if the patient brought it up.

*I believe it’s never happened … having a patient with only gout, and visiting only for that. It’s rather kind of about diabetes, heart failure or kidney disease and then they also claim a pain from the foot or … and then you have to make time for that as well … (focus group 5)*

#### Complexity of diagnostics and preventive treatment

Gout care was characterised by complicating factors concerning diagnostics, ULT titration and self-care, and it was a challenge to adapt the care according to patient preferences.

The physicians were described as mainly responsible for gout care, while nurses administered contacts with the patients. The nurses could assign a physician to renew prescriptions for analgesics and ULT and asking patients about the use of pain relief during nursing assessment, but gave no advice regarding pharmaceutical treatment. Physicians raised concerns about diagnosing gout. Several factors complicating diagnosis were mentioned, such as when the inflammation engaged the “wrong” joint (not a peripheral one), not being able to perform crystal analysis from the inflamed joint due to a lack of equipment, the urate level occasionally being low during a flare or the fact that the urate level can be high despite the absence of gout. The fact that some drugs can cause or aggravate gout was also mentioned as a complicating circumstance.

Participants mentioned that it could be a problem if a patient used painkillers and tried to self-diagnose to relieve their flares before seeking help from healthcare. If the patient was already convinced it was gout, this could lead to the physician not putting enough effort into getting the diagnostics correctly performed.

No standardised procedures were described and there was significant variation in the gout care at the different PHC units. Some of the physicians mentioned that the national recommendation for gout treatment was helpful, but some did not know about its existence and others did not use it, referring to the fact that ULT titration was too time consuming.

*… no, you start with 100 mg and follow up the with blood samples. It’s tinkering, which is why we don’t do it. Maybe. I mean, even if you’ve read how it should be done …* (Focus group 9)

The current practice for prescription and follow-up of ULT ranged between not doing it at all to performing treat-to-target titration. The same variability applied to providing information about the disease and treatment, and with the delivery of lifestyle advice.

Managers addressed the importance of care and treatment in general, and gout care specifically, being dependent on patient preferences through, for instance, patients’ narratives, their expressed needs and seeing patients as the main asset in care and treatment. Treating patients with multi-morbidity was viewed as a core mission for PHC and was seen as challenging when there was a lot to take into account.

#### Nurse-led care; a favourable organisational model when time allows it

This category represented the conditions for nurse-led gout care.

Participants claimed that nurse-led care usually paid off by generating enhanced quality of care, and strengthened the unit, especially because at times they are dependent on temporary physicians. Since it is a preferable way to organise care for other diagnoses it was considered to be applicable to treating patients with gout. There were positive experiences of giving extra responsibility for certain tasks to “assistant nurses” if they received adequate support. A problem when transferring responsibility from physicians to nurses was their already limited time, since they had already taken over some other responsibilities, for example blood pressure control and follow-up of diabetes patients. It was also pointed out that, if they had time, not only nurses but also other professional categories could be engaged when new interventions were implemented or care was reorganised.

*You can see that the nurse-led receptions often go better. So you reach target values ​​to a greater degree than if a doctor, because … .and I think that it’s sort of built into the nature of things. A patient comes to her doctor, and the doctor says, “but now we’ll talk about your gout” [but the patient says] “Yes, but yesterday this happened, which I want to talk about now”.* (Focus group 7)

### Recipients

#### A low-priority condition with acute flares

This category is related to the way in which participants described gout as minor or having lower priority than other diseases, limited to the episodes of acute flares and partially stigmatised due to being considered self-inflicted.

Participants explained, in many ways, that they see gout as a non-severe disease, how it is a problem when the patient is having a flare but not in between. Expressions such as “not important”, “not life-threatening”, “benign trouble”, “infrequent disease” and “subordinate” were used. Some of the participants considered treating gout-related pain to be enough. Other words that were used to describe gout, were “uncomplicated” and “treatable” when patients had the characteristic agonising pain. Symptoms not related to pain were seen as rare. A comparison with having an acute infection was made:

*Gout is kind of like having a cold I think, it’s only gout, and then you treat it and so on …* (Focus group 2)

Gout being a low-priority condition was also referred to when discussing diagnostics. Several physicians and nurses claimed that it did not matter whether a correct diagnosis was made; the most important issue was still helping patients to ease their pain.

*Sometimes the gout diagnosis is left out but it doesn’t matter when they don’t come back with their problem* (Focus group 2)

As a consequence, gout diagnosis was described as sometimes being performed casually, for instance on unspecified arthritis. Participants talked about gout having a history of being a stigmatising welfare disease associated with an unhealthy lifestyle, such as high alcohol consumption and overconsumption of meat. Patients with overweight multi-morbidity and male gender were identified as overrepresented.

#### Variation in knowledge about gout and belief in preventive treatment

This category sums up participants’ narratives about their inadequate knowledge about and belief in preventive ULT, broad knowledge regarding pain relief during acute gout flares and inconsistent knowledge about the kinds of support used to treat patients with gout.

Gout was described as both a well-known and an unknown disease, both among healthcare professionals and in society in general.

*Did hardly know that there was something called gout before, when I worked with surgery at the hospital, those patients didn’t have it (Focus group 2)*

A well-known aspect of gout was the characteristic pain, most often in the big toe. Gout care was perceived as organised around symptom relief, which all participants were knowledgeable about.

There was a lack of consensus among the physicians and nurses about what knowledge support systems to use when in need of more knowledge. The participants named a range of different knowledge support systems, software and websites, but were not unanimous within a specific unit or region. A need for increased knowledge among health professionals on gout and its treatment was expressed from a manager’s perspective, and concerns about the difficulties that arise due to the inconsistent use of different knowledge support systems.

The participants’ understanding of gout care was that it has remained unchanged for many decades, a fact that made it less important to put effort into creating better information or looking for up-to-date recommendations.

There was a lack of motivation to prescribe ULT and uncertainty regarding the importance of preventive drug use in between and during gout flares. Not everyone saw the necessity of motivating patients to use ULT according to recommendations.

*Neither we nor the patient feel that gout is such a troublesome thing, … thus … no exactly, it’s not so difficult. For the patient, it’s a problem during a flareup, in between you don’t care so much* (Focus group 9)

Additional arguments for not prescribing ULT were related to negative side effects, their perception of patients’ unwillingness to take medications and expectations of low compliance. Only a few physicians did not consider it difficult to motivate the use of ULT due to the characteristic pain during flareups, the development of tophi and the negative impact on the kidneys.

*I’m pretty … I actually do believe in the treatment. I’m not sure if I’m wrong … you can do good … prevent really well. That’s how I usually present it to the patient* (Focus group 6)

Some physicians and nurses described lifestyle guidance as meaningless and not contributing to changes for the individual. Uncertainty concerning lifestyle advice was reported, and explained by the fact that the scientific evidence was not seen as particularly strong.

### Context

#### A holistic but fragmented responsibility with limited resources

This category is about PHC units having too large an assignment in relation to their resources and how this influenced their responsibility for gout care.

Managers, physicians and nurses agreed that PHC is responsible for gout care. However, there were narratives of a lack of interest and insufficient support from specialist care when consultation was necessary regarding diagnostics or treatment, and the gap between PHC and specialist care was perceived as too wide.

*-But gout feels like it’s our duty (participant 1)*

*-It’s a primary healthcare diagnosis, yes … (participant 2)*

*-Rheumatologists in this region don’t expect any referrals on gout, I think, it would be someone unique, very hard to treat, or so on … (participant 1)* (Focus group 6)

Another aspect that the physicians and nurses assumed to affect gout care in PHC was the fact that patients with gout have comorbidities that are often treated in a specialist clinic. Having contact with specialist care, for whatever reason, was perceived as sometimes intervening in the healthcare managed by the PHC. Several physicians blamed their lack of experience of gout care on the lack of opportunities to follow up their patients. This was partly due to their contacts with other caregivers and left them with little experience of treatment effects, which fragmented the holistic responsibility for gout care.

The fact that person-centred care is a priority at the national level was an incentive for some of the participating managers to support holistic care, as opposed to fragmented, disease-specific care. The priority was to adapt care and treatment to the person, not to a specific diagnosis. One of the managers expressed concern related to organising PHC based on diagnosis, such as gout, fearing that it might lead to reducing the ambition to provide person-centred care.

*Person-centred care as I understand it means not to divide…we’ve been focusing a lot on the organisation created from the needs of the workplace and depending on a specific diagnosis. As a consequence, we create downpipes in primary care instead of gutters, where it’s … we don’t have any other problems, only gout* (Manager 3)

All PHC units had difficulties with the staffing situation, which caused care management problems of different kinds, including the care of patients with gout. Physicians and nurses experienced insufficient time to fulfil their responsibilities, such as keeping up to date with scientific knowledge and providing information to patients in a pedagogical manner. Temporary physicians were identified as a possible barrier to the quality of care, sometimes complicating the follow-up for patients with gout and other diseases.

Managers described the number of obligations placed on their PHC units as having a negative impact on quality of care. In addition, economic considerations often became a priority, leading to insufficient and fragmented quality improvement efforts.

#### Adopting new evidence requires supportive strategies and motivation

This category describes an awareness of what is necessary for successful implementation, including overcoming the lack of common routines regarding both gout care and the implementation of new practices.

The regions’ internal guidelines for gout care were generally considered difficult to find, with a few physicians and nurses not knowing that they existed, and they were regarded as providing insufficient support for high-quality care. Similarly, it was described as challenging to implement new practices without supporting structures, such as routines that facilitated such processes, leaving the responsibility on each individual to manage change. Managers as well as physicians and nurses confirmed the absence of such routines embracing new guidelines.

Participants referred to implementation as a complex process using words such as “process oriented” and included leadership, communication at team level and follow-up of the outcomes of potential changes as important components involved in adopting new routines. Activities used to support change processes were workplace meetings, planning days for the entire team, E-mail and the use of digital communication for education, and digital reminders in the medical journal.

Physicians and nurses described their managers as receptive, listening and creative when taking on tasks for improving care. The managers discussed “a spirit of change” and willingness to assimilate new knowledge when describing the culture in their units, which was viewed as supportive in implementation processes.

*There is a strong drive among nurses and doctors to absorb new knowledge and new ways of working* (Manager 5)

Additional support factors that increased the motivation of physicians and nurses to adopt a change were if it improved health among patients and provided care that improved cost-effectiveness.

#### Factors related to national systems and recommendations

This category deals with the lack of organisational support for booking visits and interpreting laboratory results.

Restrictions on keeping waiting lists in PHC, except for patients with certain diagnoses, was an aggravating circumstance when patients with gout were not allowed on the waiting list. This affected gout care, making it more difficult to treat it according to recommendations in terms of treat-to-target titration. However, all the participants agreed that keeping waiting lists on multiple patient groups would be impossible, and would create an excessive burden for PHC. Only a few patient groups were regularly kept on waiting lists: those with diabetes, chronic obstructive pulmonary disease, heart failure, dementia and children with special needs.

The fact that laboratory reference intervals for urate did not meet gout treatment target levels was by physicians mentioned as a possible inhibitory component for initiating preventive ULT. Electronic reminders linked to individuals diagnosed with gout were discussed as a support mechanism that would facilitate the care of patients with gout.

## Discussion

### Main findings

This is the first study in Scandinavia to describe factors influencing gout care and conditions for nurse-led gout care based on national treatment recommendations. The findings categorized under the i-PARIHS [18] construct “innovation” indicate that contacts due to gout were patient-initiated and characterised by some complexity with diagnostics and preventive treatment. Nurse-led care was described as being connected with positive experiences in PHC in general. Several important aspects related to the construct “recipients” were described as influencing gout care, such as a low belief in prevention and a view of gout as a low-priority condition, but with a broad understanding of the treatment of acute gout flares. Connected to the construct “context” was the agreement that PHC has a holistic responsibility for gout care, but organisational issues, heavy workload and shortage of staff challenged this ambition. Positive attitudes towards improvement work and awareness of factors of importance for successful implementation were also identified.

### A low-priority disease

The analysis showed variations in knowledge about gout and gout care, a lack of belief in preventive ULT and also a view of gout as lifestyle related and often self-inflicted. The overall view of gout as a second-class or low-priority disease and the varying levels of knowledge are not unique to the Swedish context. Previous studies from the perspective of both healthcare professionals and patients confirm these findings with reference to physicians’ lack of knowledge about both the causes behind gout and prevention. These studies, together with our results, emphasise the low levels of awareness about the risks to a patient with gout who persists on high urate levels [23, 24]. A comparison with other illnesses where preventive treatment is available is relevant. Migraine, similarly to gout, involves recurring pain flares that it is possible to prevent with medication [25]. As with gout, there is extensive variation regarding the prescription of preventive migraine treatment [26]. Considering the results of our study, one might assume that the description of a disease as not being a direct threat to life entails an increased risk that preventive treatment will be given low priority.

### Nurse-led care focusing on proper motivation

The findings indicate hesitation to initiate preventive treatment, partly with reference to participants’ perception that patients are generally unwilling to take drugs. This perception is not supported by the five-year follow-up by Abhishek et al. that evaluated nurse-led care for patients with gout [27]. This nurse-led care included individualised education and shared decision-making, and the findings showed high levels of adherence to preventive treatment among patients. Furthermore, a Swedish study examining patients’ willingness to consider preventive treatment in general concluded that information on Delay of Event (the time without events caused by a disease), together with shared decision-making, can be valuable in motivating patients to adhere to preventive treatment [28].

Nurse-led care was described by the participants in our study as a familiar way of organising PHC and known to be effective in terms of enhanced quality of care and being a stabilising factor when physicians were only temporary. At the same time, the participants in our study were reluctant to place a greater burden on nurses. A review of interventions to improve ULT for patients with gout concluded that nurse-led care is the most effective approach, referring to empowering the patient through education, follow-up and illness perception [29].

Nurse-led care for patients with chronic conditions is common in Swedish PHC and a recently published report emphasises that multi-professionalism characterises Swedish PHC in comparison with other European countries [30]. Furthermore, these authors recommended a reorganisation shifting responsibility from physicians to other professionals, such as nurses. The report also states that there is a need for improvement regarding the care and treatment of individuals with chronic diseases and special needs. Hence, a shift of responsibility for gout care to nurses would be in line with the desired way to organise care for chronic diseases in general. Nurse-led care, including education, motivating information about delay of events and shared decision-making, might be beneficial in terms of supporting patients’ understanding of their conditions and the effects of preventive treatment, thereby increasing their motivation for it.

### Person-centred care

It has been suggested that using a person-centred approach in PHC is crucial for the development of sustainable healthcare [31, 32]. Person-centred care originates in an ethical standpoint guiding actions and entails the patient narrative, a partnership between patient and professionals (e.g. shared decision-making) and documentation as core components [33]. Individualisation and shared decision-making are important parts of the nurse-led care model referred to above [34].

The managers in our study feared that care organised around a specific diagnosis, such as gout, might lead to less holistic care. This was pointed out with reference to the importance of an orientation towards more person-centred PHC. The managers’ willingness to prioritise person-centred care is confirmed by the fact that one of the three regions included in this study, together with 12 other regions in Sweden, has taken a decision to allocate resources to the development of PHC in a person-centred direction [35]. Accordingly, to achieve better care for patients with gout as well as other chronic diseases, a shift towards person-centred care is preferable. This must include all diagnoses when co-creating care and treatment with a patient, thus avoiding focusing on a specific diagnosis only.

### Implementation of recommendations

Our results indicate that the national treatment recommendations for gout have not been sufficiently disseminated or implemented since being published in 2016. According to Sandstrom et al., there is a lack of standardised procedures in Sweden to support the implementation of new guidelines in PHC [36]. Another reason could be clinical inertia, a phenomenon that has been connected with undertreatment of patients with gout in previous research [37]. Clinical inertia means that a need to initiate the best available treatment is recognised but at the same time denied, due to uncertainty concerning the outcome of the decision [38]. This is clearly in line with the findings of our study, which point to both the existing uncertainty about evidence for lifestyle advice and a lack of belief in preventive treatment for gout.

Our study shows an awareness among managers of the factors motivating physicians and nurses at PHC units for new practices, such as interest in and understanding of the innovation. Theories on implementation identify the “relative advantages” of a new practice (i.e. that the new practice is beneficial compared to existing treatment) as crucial, affecting the motivation and likeliness to adopt a change [38]. However, the motivation to deliver preventive treatment to patients with gout was, as discussed above, sometimes lacking. Regardless of the reason behind this non-conviction, a low belief in preventive treatment negatively affects the delivery of care based on the associated recommendations [37]. Hence, implementation efforts with the purpose of improving gout care in PHC should embrace the relative advantages compared to retaining the old way of approaching gout care. By addressing the inadequate knowledge, the feelings of complexity and lack of belief in preventive treatment, the view that it is less important to prevent gout than other diseases could become less prevalent. A multicomponent strategy for implementation of recommendations and changed care processes have been proven to have a positive outcome on clinical practice [39, 40]. Such a strategy will most likely be needed to address the identified challenges of this study and could include workshops, paper-based educational materials such as checklists and reminders [41] and different educational strategies including educational meetings [40].

The positive attitudes towards nurse-led care and improvements from this study results might be used in a future implementation. It could also be important to clarify the ways in which time given to prescription, titration and motivation to adhere to preventive treatment can reduce the current time and visits spent in managing acute pain. A systematic review of qualitative evidence on barriers and facilitators for task shifting in primary healthcare, physicians to nurses, stated that both professions valued the shifting. However, they also remarked the importance of maintaining a close partnership between them [42] and future implementation of task shifting must consequently make efforts to safeguard well-functioning collaborations and support the development of new forms of collaboration.

### Strengths and limitations

A considerable strength of this study is the use of focus group discussions for data collection, which is a method perceived to generate rich data. This is supported by the literature, which suggests that focus groups as a method of data sampling decreases the potential negative influence of researchers’ authority, thereby increasing the chances of gathering rich data and a deeper understanding of the phenomenon under study [20]. The focus group discussions and individual interviews were led by the same moderator using the same themes, thus strengthening the likelihood of all interviews having a similar structure. Other strengths are the representation of differences (e.g. unit size, geographical conditions) at the included PHC units and the high number of participants, as well as the inclusion of managers, which enabled the inclusion of both clinical and managerial perspectives in the analysis. This broad variety in representation can generate transferability to other, similar contexts. The i-PARIHS framework was used for the analysis, but not for creating the themes that guided the group discussion and interviews. Thus, the discussions were not limited by the boundaries of the framework. The large number of sub-constructs within the i-PARIHS was experienced as fragmenting the content of the data in an unfavourable manner; therefore, they were only used as guidance (additional file 2), to keep track of data suitable for each main category during the initial process of analysis. The entire content of the collected data did however fit into the matrices used in the deductive analysis, nothing had to be analysed separately. Furthermore, authentic citations, together with results tables, have been used to increase trustworthiness.

A limitation of the study might be connected to the purposive sampling of nurses and physicians. Managers were told to select physicians and nurses, in order to obtain variety in genders and professions, but we know nothing about those not asked to participate, which involves the risk of possible selection bias. Although the benefits of choosing focus groups for data sampling met the aim of our study, a potential limitation connected to this choice concerns the mixed professions in the focus groups related to hierarchies within a group, such as: nurse-physician, male-female or beginner-senior. Despite this, our assessment is that knowledge differences and dissidents emerged.

## Conclusion

This study, investigating the perspectives of PHC professionals, identified several factors that influence current gout care, as well as the conditions and barriers for a possible future nurse-led gout care. We have identified inadequate knowledge regarding the value of preventive treatment and how to initiate this treatment as factors that need to be addressed in order to achieve improved care and treatment for patients with gout. Further, we also identified positive attitudes towards nurse-led care and towards practice development of clinical practice, which may facilitate the implementation of nurse-led gout care.

## Supplementary Information

**Additional file 1.**

**Additional file 2.**

**Additional file 3.**

## Data Availability

The datasets analysed during the current study are not publicly available due to the fact that this was not agreed with the healthcare professionals providing the data, but are available from the corresponding author on reasonable request.

## Abbreviations

PHC: Primary healthcare
ULT: Urate-lowering treatment
