# Supplementary material for: Professionals’ perspectives on existing practice and conditions for nurse-led gout care based on treatment recommendations: a qualitative study in primary healthcare
Source: BMC Prim Care. 2022 Apr 7;23:71. doi: 10.1186/s12875-022-01677-z (PMC8988383; doi:10.1186/s12875-022-01677-z)
Supplement: Supplementary file 1 — Additional file 1. [file 12875_2022_1677_MOESM1_ESM.docx]

Main topics

Tell us about your experiences of gout and patients with gout**.**

What is gout?

Who has gout?

Are there common features between patients with gout?

According to your experiences, how is gout affecting daily life?

Those you have met with gout, how much do they know about their disease?

Tell us about the care and treatment of patients with gout, the support and efforts patients with gout participate in at your primary healthcare unit.

Treatment - initiation, follow-up?

Tell us about patient participation in their treatment, planning and follow-up.

What are your thoughts on what affects the care people with gout receive today? (time, resources, knowledge, organization, guidelines, priorities, employees, leadership)

What do you think about the similarities and differences of interventions and support for patients with gout compared to other diagnostic groups (diabetes, heart failure)

Treatment guidelines, what do you think about them, are they important? How do you think about different guidelines, what influence do they have?

1. Tell us about contextual conditions for, and experiences of, implementation of new practices in general

What interventions as support and information do you think could help patients follow the treatment you recommend?

Do you see any difficulties or challenges with this? (time, resources, knowledge, organization, leadership)

When it comes to new methods, treatments or guidelines that require a change in working methods - how do you proceed to achieve this?

How do healthcare professionals want to gain knowledge about evidence-based guidelines and knowledge about new ways of working?

1. Tell us about your views on and potential of nurse-led care in general (at the same time the focus group get a short information about a British study using nurse-led care treating patients with gout)

In what aspects is nurse-led care a suitable way of organising treatment in primary health care?

How could something similar as in UK be incorporated here - what would it look like? What would need to change?
