# Supplementary material for: Professionals’ perspectives on existing practice and conditions for nurse-led gout care based on treatment recommendations: a qualitative study in primary healthcare
Source: BMC Prim Care. 2022 Apr 7;23:71. doi: 10.1186/s12875-022-01677-z (PMC8988383; doi:10.1186/s12875-022-01677-z)
Supplement: Supplementary file 2 — Additional file 2. [file 12875_2022_1677_MOESM2_ESM.docx]

|  | **Generic cathegory**, characteristics of the innovation, sourcing and applying available research evidence to inform the innovation | **DEFINITION** |
| --- | --- | --- |
|  | Underlying knowledge sources | A description of existing evidence, credibility and trustworthiness with the recipients. |
| What to be implemented: a broad combination | Clarity | Describes how people can easily and clearly see what is proposed for the process of patient care |
| of research evidence, knowledge and practice | Degree of fit within existing practice and values | Describes who will be affected by the innovation and how. Also includes how much consensus there are around the evidence, individually, within and between different groups affected, if the respond on the innovation will be depending on group(patients, managers, professional groups) asked. |
|  | Degree of novelty | Describes changes required for adapting the innovation: in care processes, peoples way of thinking, knowledge to be transformed. |
|  | Usability | Describes if the evidence are packed and accessed in a usable form. |
|  | Relative advantage | Describes if the innovation offers any advantages compared to the current way of doing things according to both facilitators and those affected by the innovation. Includes cost-effectiveness |
|  | Trialabilty | Describes whether the innovation is possible to try out in a small scale before implementing |
|  | Observable results | Describes how the innovation will lead to results possible to observe by the recipients, such as patient experience and greater efficiency on provision of care |
| **Main construct:** RECIPIENTS | **Generic cathegory**: characteristics of the recipients to be considered in the implementation, how they will respond to the changes required as individuals and as team | **DEFINITION,** the questions “want to?” and “can do?” are central when defining the characteristics of the recipients. The impact from the team and individuals to support or resist the innovation. |
| Staff, services, patients to be involved in and | Motivation | Describes the motivation that resist or support the innovation |
| affected by the implementation | Values and beliefs | Describes how the recipients’ values and beliefs are consistent with the proposed change/innovation. |
| As teams and individuals | Goals | Describes how the existing goals are resisting or supporting the innovation suggested. |
|  | Skills and knowledge | Describes the current level of skills and knowledge and if/how that level has to be complemented to be in accordance with the underlying knowledge sources included in the innovation |
|  | Time, resources and support | Describes the time, resources and support available for support or resist the innovation |
|  | Local opinion leaders | Describes the ways in which the local opinion leaders support or resist the innovation |
|  | Collaboration and teamwork | Describes the existing collaboration and teamwork and how that can support or resist the innovation |
|  | Existing networks | Describes networks available that can support or resist the innovation |
|  | Learning environment | Describes how the learning environment can support or resist the innovation |
|  | Power and authority | Describes how the existing power and authority can support or resist the innovation |
|  | Presence of boundaries | Describes professional boundaries and network present that can resist or support the innovation |
| **Main construct**: INNER CONTEXT | **Generic cathegory:** characteristics of the context to be considered in the implementation | **DEFINITION**, the immediate work environment of the recipients |
| **-local level** | Formal and informal leadership support | Identification of the leaders, informal and formal, and their likeliness to support the change/innovation. In what extent do the formal leaders work facilitative and democratic? |
| The setting or unit set up for implementation | Culture | Identification of the character of the local culture and if it is supportive for innovation and change: do staff feel actively involved in decision that involves them, do patients and staff feel valued, are new ideas trusted to be introduced? |
|  | Past experience of innovation and change | Influences of past experiences of participating in changes, supporting or resisting a new change taking place |
|  | Mechanisms for embedding change | Structures and processes that integrate a change into routine practice |
|  | Evaluation and feedback processes | Structured processes at the setting that evaluate and reconnect results from a change to the recipients |
| **-organisational level** | Organisational priorities | In what extent the innovation is aligned with organisational priorities |
| The organisation in which the unit | Leadership and senior management support | In what degree are the leadership and management organised in a hierarchical way (tends to have an association with prolonging and complicating the implementation process) |
| is located | Culture | Culture within the organisation concerning the allowance (managing and responding) of changes/new innovations historically |
|  | Structure and systems | Structure and systems supporting or resisting change |
|  | History of innovation and change | History of innovation and change within the organisation resisting or supporting a new change |
|  | Absorptive capacity | Degree of how knowledge is used within the organisation to reflect learn |
|  | Learning networks | Do the organisation continuously participate in different learning network to monitor and evaluate performance |
| **Main construct:**  **OUTER CONTEXT** | **Generic cathegory** | **DEFINITION** |
|  | Policy drivers and priorities | In what extent the innovation is aligned with the wider health system strategic priorities |
| The wider | Incentives and mandates | Existence of incentives and mandates that support or resist the innovation. (Ex. pay for performance) |
| health system in which the | Regulatory framework | Existence of regulatory framework supporting or resisting the innovation |
| organisation is based | Environmental (in)stability | Character on the wider health system to be stabil or instabil. |
|  | Inter-organisational networks and relationships | Existence of inter-organisational networks that can support the innovation |

*A description of the planned implementation process and where in the process the present study belongs*

I-PARIHS framework Implementation process; evidence based practice

Context: primary health care in three regions in the middle of Sweden

Facilitation: **examining the pre-requisites for conducting an implementation of a complex innovation: interviews with HCP** and patients. Selecting change ideas possible to adapt in the considered units. Preparations of necessary education material and lessons for both HCP and patients.

Recipients: HCP on primary health care units from the tree involved regions. Patients with gout from the same context.

Facilitation: A dedicated facilitator is named. Modification of the innovation with adjustment to the specific settlement on each unit takes place

Innovation: A new way of working with care and treatment of persons with gout is tested by HCP in an “everyday” primary health care environment during a period of 1 year.

Evaluation of the INNOVATION in a feasibility study/pilot study.

Are there recognised improvements on quality of care and treatment for persons with gout?

Facilitation: Modification of the innovation according to the results in the feasibility study.

Innovation: A case study takes place implementing the INNOVATION in an “everyday” primary health care environment.
