# Supplementary material for: Professionals’ perspectives on existing practice and conditions for nurse-led gout care based on treatment recommendations: a qualitative study in primary healthcare
Source: BMC Prim Care. 2022 Apr 7;23:71. doi: 10.1186/s12875-022-01677-z (PMC8988383; doi:10.1186/s12875-022-01677-z)
Supplement: Supplementary file 3 — Additional file 3. [file 12875_2022_1677_MOESM3_ESM.docx]

| **Constructs** | **Categories** | **Sub-categories** |
| --- | --- | --- |
| **Innovation** | Complexity of diagnosis and treatment | Clinical practice dependent on physician involved |
|  |  | A diagnostic dilemma |
|  |  | Prevention too time-consuming |
|  |  | Self-care common^1^ |
|  |  | Emphasizing the importance of treatment depending on patient preferences^2^ |
|  | Nurse-led care; an advantageous organisational model when time allows it | When time allows, nurses can be assigned with new responsibilities |
|  |  | Advantages with nurse-led care |
| **Recipients** | A low-priority acute condition | Minor to other diseases |
|  |  | Male and self-inflicted disease |
|  |  | Painful flares, otherwise mild and unimportant |
|  | Variation in knowledge about gout and belief in preventive treatment | Both well-known and un-known |
|  |  | Uncertainty on the need of motivating preventive treatment^2^ |
|  |  | Broad knowledge on pain relief^1^ |
| **Context**, inner (local and organisational) and outer | A holistic/principal/main responsibility but fragmented and with limited recourses | Responsibility for gout lies on primary health care |
|  |  | An aggravating organizational gap between specialist and PHC |
|  |  | Person centred care has become a national priority^2^ |
|  |  | Deficiency in competence provision^1^ |
|  |  | Endless obligations is a holdback according to managers |
|  | Adopting new evidence demands supportive strategies and motivation | Failed support system |
|  |  | Changes demands motivation and a culture of improvement |
|  |  | A new routine demands a routine |
|  | Aggravating circumstances related to systems and recommendations | Normal s-urate interval not compatible with recommendations^2^ |
|  |  | Restrictions aggravates follow-up appointments |

^1^ Only expressed by nurses and physicians ^2^ Only expressed by managers
